# Parents’ and guardians’ acceptability of COVID-19 vaccination for children in Ghana: An online survey

**DOI:** 10.1371/journal.pone.0272801

**Published:** 2022-08-29

**Authors:** Frank Kyei-Arthur, Sylvester Kyei-Gyamfi, Martin Wiredu Agyekum, Grace Frempong Afrifa-Anane, Bernard Akyeampong Amoh

**Affiliations:** 1 Department of Environment and Public Health, University of Environment and Sustainable Development, Somanya, Ghana; 2 Department of Children, Ministry of Gender, Children and Social Protection, Accra, Ghana; 3 Institute for Educational Research and Innovation Studies, University of Education, Winneba, Ghana; 4 Centre for Migration Studies, University of Ghana, Accra, Ghana; Flinders University, AUSTRALIA

## Abstract

Few studies have examined the intentions of parents and guardians to vaccinate their children younger than 18 years against COVID-19 in Ghana. Parents are the decision makers for children younger than 18 years; therefore, we examined parents’ and guardians’ intentions to accept the COVID-19 vaccines for their children. An online survey was conducted among 415 parents and guardians in Ghana. The Statistical Package for Social Sciences version 25 was used to analyse the data. We found that 73.3% of parents/guardians would allow their children to be vaccinated against COVID-19. The binary logistic regression analysis shows that parents/guardians with Senior High School education, those who believed COVID-19 could not be cured, and those who agreed and those who neither agreed nor disagreed with the statement “once the vaccine is available and approved, it would be safe” were less likely to accept COVID-19 vaccine for their children. Also, parents/guardians who neither agreed nor disagreed that “the best way to avoid the complications of COVID-19 is by being vaccinated”, those who agreed that “I am of the notion that physiological/natural community is better compared to vaccine-induced immunity” and “I believe the vaccine programming may be likened to the new world order” were less likely to accept COVID-19 vaccine for their children. There is a need for public health practitioners to intensify education on the benefits and side effects of COVID-19 vaccines, as well as provide regular and up-to-date information about vaccines’ safety to parents and guardians.

## Introduction

Most countries are experiencing the fourth wave of the COVID-19 pandemic, and Ghana is no exception [1]. Ghana experienced a surge in its COVID-19 cases after December 13, 2021. Between December 1 and 8, 2021, COVID-19 cases increased by 46 cases, while it increased by 9,257 cases between December 23 and 30, 2021 [2].

Vaccination against COVID-19 is one of the effective measures to slow the spread and reduce mortality associated with it [3]. Ghana’s population is estimated to be 30.8 million, with children younger than 18 years constituting two-fifths (41.8%) in 2021 [4]. The Government of Ghana aims to vaccinate all Ghanaians against COVID-19, with an initial target of 20 million by the first quarter of 2022 [5]. Owing to this, the Food and Drugs Authority of Ghana has approved five vaccines: AstraZeneca, Sputnik V, Moderna, Johnson & Johnson, and Pfizer, for the use of the entire exercise across the country [6].

Han et al. [7] noted that Pfizer and CoronaVac vaccines were safe for children below 18 years. The Serum Institute of India Pvt Limited, and Moderna, the companies which manufacture AstraZeneca and Moderna vaccines, respectively, are conducting clinical trials to develop COVID-19 vaccines for children younger than 18 years [8]. Countries like the United States of America (USA), the United Arab Emirates, Cuba, and China have approved COVID-19 vaccination for children younger than 18 years [9]. In the USA, the Food and Drug Administration and Centers for Disease Control and Prevention have approved the emergence use of the Pfizer vaccine for children aged between 5 to 11 after initially approving it for children aged 12 and older [9, 10].

Ghana’s current COVID-19 vaccination programme cover persons aged 15 and older [11]. Previous studies have established that children are less likely to develop severe symptoms of COVID-19 and are less likely to spread COVID-19 than adults [7, 12]. Nevertheless, children in Ghana have been heavily impacted by COVID-19 infection [13]. Child Rights International [14] has estimated that about 2,180 children younger than 18 years were infected with COVID-19 between March 11 and November 9, 2020, in Ghana. Likewise, approximately 2,323 students from 363 primary, secondary, and tertiary educational institutions across the country were infected with COVID-19 after reopening educational institutions in January 2021 [15].

Children younger than 18 years are regarded as minors. They are, therefore, legally required to have decisions made on their behalf by their parents, guardians, and other duty bearers in charge of their education, social welfare, and health [16–18]. While research on parents’ and guardians’ intention to vaccinate their children against COVID-19 is at an advanced stage in North America (e.g., the United States of America and Canada), Asia (e.g., China), and Europe (e.g., Turkey, Germany, and England) [19–22], very little attention has been given to the issue in sub-Saharan African countries, including Ghana. For example, a study among parents in China found that about 73% of parents were willing to vaccinate their children against COVID-19 when vaccines become available [23]. A similar study among adults in the United States found that 73% of adults intended to vaccinate their children against COVID-19 [24]. Among the factors that influence parents’ and guardians’ acceptability of COVID-19 vaccination for children are parents’ age, educational level, and knowledge about COVID-19 preventive measures. Other factors include household income levels, employment status, the number of children parents have, children with no chronic illness, worry about child getting COVID-19, confidence in vaccine safety, and belief in importance of vaccines [19, 20, 24–26].

In sub-Saharan Africa, few studies have examined the intentions of parents and guardians to vaccinate their children against COVID-19. For example, Carcelen et al.’s [27] study among caregivers with children aged six months to 5 years in Zambia found that 9 out of 10 caregivers (92%) were willing to vaccinate their children against COVID-19. There is thus a dearth of studies on parents’ and guardians’ intention to vaccinate their children against COVID-19 and factors that influence their intention in sub-Saharan Africa, including Ghana. Therefore, this study sought to estimate the prevalence of acceptability of COVID-19 vaccination for children among parents and guardians in Ghana. It also examined the factors influencing parents and guardians intentions to accept the COVID-19 vaccination for their children. Knowing and examining the intentions of parents and guardians to vaccinate their children against COVID-19 will help researchers and public health practitioners initiate and develop suitable measures to promote the uptake of COVID-19 vaccination for children in the country.

## Materials and methods

### Study design and sampling procedure

This study is a cross-sectional one conducted among parents and guardians in Ghana using convenient and snowballing sampling techniques. Google Form was used to design an online self-administered questionnaire. The online survey was shared on WhatsApp, Meta (Facebook), and Twitter platforms. It was also shared via email with relatives, friends, and colleagues. Parents and guardians were encouraged to share the survey link with relatives, friends, and colleagues who were also parents or guardians. Inclusion criteria for the study include: respondent being a Ghanaian parent or guardian aged 18 and older who has biological or foster children aged below 18 in their care and residing in Ghana at the time of the survey. Also, Ghanaian parents or guardians aged 18 and older with no biological or foster children aged below 18 in their care were excluded from the study.

### Study setting

Ghana is a lower-middle-income country located in West Africa. Ghana shares a boundary with Burkina Faso in the North, the Gulf of Guinea in the South, Togo in the East, and Cote d’Ivoire in the West. Ghana has 30.8 million people and 16 administrative regions as of 2021. The current population comprises 50.7% female and 49.3% male [28]. Ghana covers a land area of 238,533 sq. km, and the Greater Accra region is its capital. About 17.7% of people in Ghana reside in the Greater Accra region, the most densely populated region (1,678.3 people per sq. km) in Ghana [28]. Currently, the Greater Accra region is the most populous in the country, growing from 16.3% in the last census (2010) to 17.7% in the latest (2021), while the population of the Ashanti Region declined from 19.4% to 17.6%.

### Data collection

The data for the study was collected over three months, ending November 2021, from parents and guardians. The online survey questionnaire covered four areas: socio-demographic characteristics, COVID-19 experience, beliefs about COVID-19 vaccines, and intention to vaccinate child/children (See S1 Questionnaire). The questionnaire had 50 questions, comprising 12 questions on socio-demographic characteristics, 22 on COVID-19 experience, 11 on beliefs about COVID-19 vaccines, and 5 on intention to vaccinate child/children.

The questions on socio-demographic characteristics, COVID-19 experience, beliefs about COVID-19 vaccines, and intention to vaccinate child/children were adapted from previous studies [3, 19, 25, 29]. However, these questions were modified in light of the Ghanaian context.

On the first page of the online survey, a brief introduction was provided, which contained the aims of the study, inclusion and exclusion criteria, and consent statement. Respondents provided written consent to participate in the study by selecting “I consent to participate in this study” in the consent statement section. Only respondents who provided written consent could fill out the Google Form. Participation in the study was voluntary. The study complied with all ethical regulations and written informed consent was obtained from all respondents. Ethical approval for the study was obtained from the Ethics Committee of the University of Environment and Sustainable Development, Somanya—Ghana (APP/RSC/0001).

### Study variables

#### Dependent variable

The dependent variable for the study was parents’ and guardians’ acceptability of COVID-19 for children. Respondents were asked, “If an approved COVID-19 vaccine became available, would you allow your child/children to be injected/vaccinated” and the responses were “Yes”, “No”, and “Don’t know”. The No” and “Don’t know” responses were recoded as “No”.

#### Independent variables

The study’s independent variables included socio-demographic characteristics of respondents, COVID-19 experiences, and beliefs about COVID-19 vaccines (See S1 Appendix). The socio-demographic characteristics of the parents and guardians were age (less than 30, 30–39, 40–49, and 50 and older); sex (male/female); religion (Christians and non-Christians); education (less than Senior High School (SHS), SHS, and Undergraduate); and marital status (never married, living together, married, separated/divorced/widowed). Other socio-demographic characteristics included employment status (currently employed and not currently employed); region of residence (Greater Accra, Central, Western, Eastern, and other regions); household wealth (more money than you need, just enough money, and less money than you need); the number of children younger than 18 years (1, 2–3, 4 and above).

Parents and guardians were asked about their COVID-19 experiences. Questions asked included whether parents/guardians have ever had a COVID-19 test; a member of their household, relative, friend, or neighbour had been diagnosed with COVID-19; the child’s chance of being infected with COVID-19 at home, community or school; whether parents and guardians changed their adherence to COVID-19 preventive protocols due to Government’s decision to provide COVID-19 vaccine; if it is possible for a healthy-looking person to have COVID-19; and whether COVID-19 could be cured.

Also, parents and guardians were asked 11 questions about their beliefs about COVID-19 vaccines. The response to each question was “strongly disagree”, “disagree”, neither agree nor disagree”, “agree”, and “strongly agree”. All responses were recoded in the same direction. “Strongly disagree” and “disagree” responses were recategorised into “Disagree” while “agree” and “strongly agree” responses were recategorised into “Agree”. The reliability of the 11 questions about parents’ and guardians’ beliefs about COVID-19 vaccines was assessed using Cronbach’s alpha coefficient. The Cronbach’s alpha score for the beliefs about COVID-19 vaccines was 0.738 which indicates it is reliable or internally consistent.

### Data analysis

The data were analysed using the Statistical Package for Social Sciences (SPSS) version 25 for Windows. Descriptive statistics, such as frequencies and percentages, were used to describe the socio-demographic characteristics of respondents, COVID-19 experiences, and beliefs about COVID-19 vaccines. Pearson’s chi-square test was also used to determine the association between parents’/guardians’ acceptability of COVID-19 for their children and socio-demographic characteristics of respondents, COVID-19 experience, and beliefs about COVID-19 vaccines. The dependent variable was dichotomous, so binary logistic regression was used to examine the factors influencing parents’ and guardians’ intentions to accept the COVID-19 vaccination for their children. All variables with a p-value < 0.05 was considered to be statistically significant.

## Results

### Acceptability of COVID-19 vaccine for children younger than 18 years

**Fig 1:**
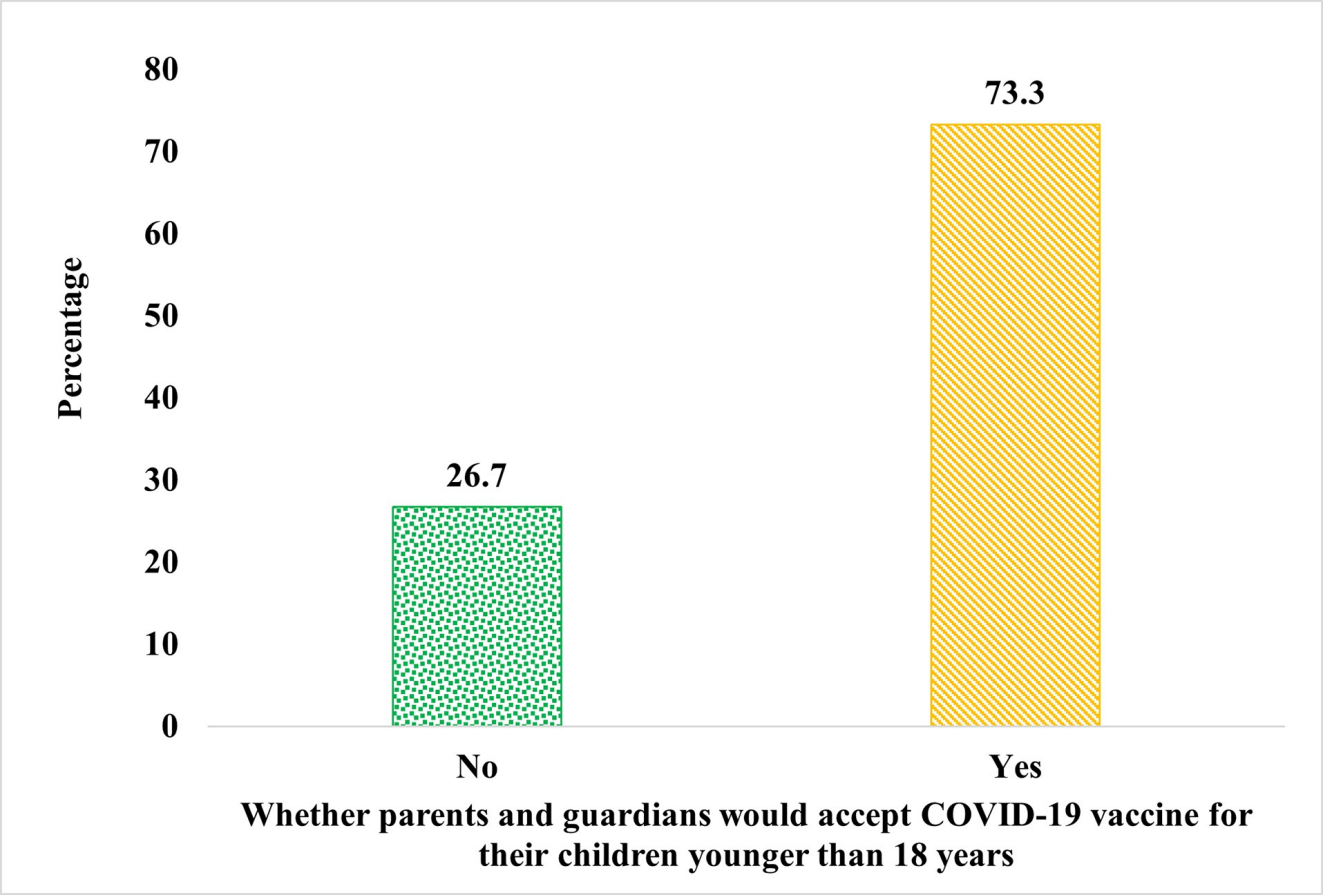
Acceptability of COVID-19 vaccine for children younger than 18 years.

A total of 415 parents and guardians were involved in this study. More than two-thirds (73.3%) of the parents and guardians indicated their acceptance of the COVID-19 vaccine for their children younger than 18 years, whiles 26.7% stated non-acceptance of COVID-19 vaccine for their children (Fig 1). The main reasons cited for non-acceptance of COVID-19 vaccine by parents and guardians included inadequate data about the safety of the new vaccine (34.3%), concern about the adverse effects of the vaccine (18.0%), prior adverse reaction to any vaccine (6.3%), and parents and guardians perceiving their children not to be at any considerable risk of developing complications if infected with COVID-19 (6.3%) (Table 1).

**Table 1:** Main reason for the non-acceptance of the COVID-19 vaccine for children.

	n (%)
Inadequate data about the safety of the new vaccine	38 (34.3)
A concern on adverse effects of the vaccine	20 (18.0)
Prior adverse reaction to any vaccine	7 (6.3)
I perceive my child not to be at considerable risk of developing complications if he/she is infected with COVID-19	7 (6.3)
A concern on the vaccine being ineffective	4 (3.6)
I am against vaccines in general	4 (3.6)
A concern of acquiring COVID-19 infection from the vaccine itself	3 (2.7)
I have not taken mine, so I will not let my child take it	2 (1.8)
I perceive my child not at elevated risk to acquire COVID-19 infection	2 (1.8)
Other responses	13 (11.7)
No response	11 (9.9)

### Socio-demographic characteristics of respondents

Regarding age, the highest proportion (39.8%) of the respondents were aged 30–39 (Table 2). Slightly more than half (50.6%) of them were females, and nine out of ten (91.6%) were Christians. In terms of education, the highest proportion of the respondents (46.7%) had undergraduate education, whiles slightly less than one-fifth (19.3%) had less than Senior High School (SHS) education. The highest proportion (86.2%; p < 0.05) of those with less than SHS education indicated their acceptance of the COVID-19 vaccine for their children than those with SHS (70.2%; p < 0.05) and Undergraduate (70.1%; p < 0.05) education. About two-thirds (63.4%) of the respondents were married, while a few (6.7%) lived together. On parents’ and guardians’ employment status, more than two-thirds (83.4%) were currently employed, and the majority (71.3%) of them resided in the Greater Accra region. A little over half (51.8%) of the respondents had less money than they would need. The highest proportion (49.4%) of them indicated having 2–3 children, while less than two-fifths (16.1%) reported having four or more children younger than 18 years.

**Table 2:** Socio-demographic characteristics of respondents.

		Acceptability of COVID-19 vaccine for children		
Variables	N = 415 n (%)	No n (%)	Yes n (%)	p-value
**Age of parent and guardian**				
Less than 30	45 (10.8)	13 (28.9)	32 (71.1)	0.208
30–39	165 (39.8)	50 (30.3)	115(69.7)	
40–49	108 (26.0)	30 (27.8)	78 (72.2)	
50 and older	97 (23.4)	18 (18.6)	79 (81.4)	
**Sex of parent and guardian**				
Male	205 (49.4)	54 (26.3)	151 (73.7)	0.854
Female	210 (50.6)	57 (27.1)	153 (72.9)	
**Religion of parent and guardian**				
Christians	380 (91.6)	99 (26.1)	281 (73.9)	0.292
Non-Christians	35 (8.4)	12 (34.3)	23 (65.7)	
**Education of parent and guardian**				
Less than SHS	80 (19.3)	11 (13.8)	69 (86.2)	0.014**
SHS	141 (34.0)	42 (29.8)	99 (70.2)	
Undergraduate	194 (46.7)	58 (29.9)	136 (70.1)	
**Marital status of parent and guardian**				
Never married	85 (20.5)	23 (27.1)	62 (72.9)	0.210
Living together	28 (6.7)	7 (25.0)	21 (75.0)	
Married	263 (63.4)	76 (28.9)	187 (71.1)	
Separated/divorced/widowed	39 (9.4)	5 (12.8)	34 (87.2)	
**Employment status of parent and guardian**				
Currently employed	346 (83.4)	88 (25.4)	258 (74.6)	0.176
Not currently employed	69 (16.6)	23 (33.3)	46 (66.7)	
**Region of residence of parent and guardian**				
Greater Accra	296 (71.3)	76 (25.7)	220 (74.3)	0.649
Central	20 (4.8)	8 (40.0)	12 (60.0)	
Western	23 (5.5)	5 (21.7)	18 (78.3)	
Eastern	30 (7.2)	9 (30.0)	21 (70.0)	
Other regions	46 (11.1)	13 (28.3)	33 (71.7)	
**Household wealth**				
More money/just enough money	200 (48.2)	50 (25)	150 (75)	0.438
Less money than you need	215 (51.8)	61 (28.4)	154 (71.6)	
**Number of children younger than 18 years**				
1	143 (34.5)	43 (30.1)	100 (69.9)	0.463
2–3	205 (49.4)	53 (25.9)	152 (74.1)	
4 and above	67 (16.1)	15 (22.4)	52 (77.6)	

p < .1*, p < .05**, p < .001***

### COVID-19 experiences

COVID-19 experiences of the respondents are seen in Table 3. The results show that about seven out of ten (73.5%) parents and guardians had never tested for COVID-19, while less than one-tenth (7.0%) had members of their household diagnosed with COVID-19. Furthermore, the majority (79.3%) of them reported that none of their relatives had been diagnosed with COVID-19. In addition, more than half (58.1%) of the respondents had none of their friends been diagnosed with COVID-19. Also, the majority (67.5%) of them reported that none of their neighbours were diagnosed with COVID-19.

**Table 3:** COVID-19 experiences.

		Acceptability of COVID-19 vaccine for children		
Variables	N = 415 n (%)	No n (%)	Yes n (%)	p-value
**Ever had a COVID-19 test**				
Yes	110 (26.5)	23 (20.9)	87 (79.1)	0.107
No	305 (73.5)	88 (28.9)	217 (71.1)	
**Member of household been diagnosed with COVID-19**				
Yes	29 (7.0)	8 (27.6)	21 (72.4)	0.916
No	386 (93.0)	103 (26.7)	283 (73.3)	
**Relatives have been diagnosed with COVID-19**				
Yes	50 (12.0)	15 (30.0)	35 (70.0)	0.518
No	329 (79.3)	84 (25.5)	245 (74.5)	
Don’t know/Can’t tell	36 (8.7)	12 (33.3)	24 (66.7)	
**Friends have been diagnosed with COVID-19**				
Yes	123 (29.6)	35 (28.5)	88 (71.5)	0.120
No	241 (58.1)	57 (23.7)	184 (76.3)	
Don’t know/Can’t tell	51 (12.3)	19 (37.3)	32 (62.7)	
**Neighbours been diagnosed with COVID-19**				
Yes	38 (9.2)	9 (23.7)	29 (76.3)	0.060*
No	280 (67.5)	67 (23.9)	213 (76.1)	
Don’t know/Can’t tell	97 (23.4)	35 (36.1)	62 (63.9)	
**Child’s chance of getting COVID-19 at home**				
Small	110 (26.5)	20 (18.2)	90 (81.8)	0.059*
Moderate	45 (10.8)	12 (26.7)	33 (73.3)	
Great	16 (3.9)	7 (43.8)	9 (56.3)	
No risk at all	244 (58.8)	72 (29.5)	172 (70.5)	
**Child’s chance of getting COVID-19 in the community**				
Small	144 (34.7)	46 (31.9)	98 (68.1)	0.069*
Moderate/ Great	61 (14.7)	10 (16.4)	51 (83.6)	
No risk at all	210 (50.6)	55 (26.2)	155 (73.8)	
**Child’s chance of getting COVID-19 at school**				
Small	102 (24.6)	28 (27.5)	74 (72.5)	0.886
Moderate	87 (21.0)	24 (27.6)	63 (72.4)	
Great	58 (14.0)	13 (22.4)	45 (77.6)	
No risk at all	168 (40.5)	46 (27.4)	122 (72.6)	
**Government’s decision to provide COVID-19 vaccine changed your adherence to COVID-19 prevention protocols**				
Yes	33 (8.0)	10 (30.3)	23 (69.7)	0.630
No	382 (92.0)	101 (26.4)	281 (73.6)	
**Possible for a healthy-looking person to have COVID-19**				
Yes	342 (82.4)	94 (27.5)	248 (72.5)	0.462
No	73 (17.6)	17 (23.3)	56 (76.7)	
**Can COVID-19 be cured**				
Yes	263 (63.4)	56 (21.3)	207 (78.7)	0.001**
No	152 (36.6)	55 (36.2)	97 (63.8)	

p < .1*, p < .05**, p < .001***

Regarding the risk of children being exposed to COVID-19 at home and in their communities, 58.8% and 50.6% of the respondents indicated that their children had no risk of getting COVID-19 at home and in the community, respectively. Approximately two-fifths (40.5%) of the parents and guardians indicated that their children had no risk at all of getting COVID-19 at school. In terms of Government decisions, most (92.0%) of them reported that their adherence to COVID-19 prevention protocols has not changed because the government decided to provide the COVID-19 vaccine. Furthermore, the majority (63.4%) of the respondents felt that COVID-19 could be cured. A higher proportion (78.7%; p < 0.05) of parents and guardians who felt COVID-19 could be cured indicated their acceptance of the COVID-19 vaccine for their children than those who felt COVID-19 could not be cured (63.8%; p < 0.05).

### Beliefs about COVID-19 vaccines

On the beliefs of COVID-19, about 61% of the respondents disagreed with the statement “once the vaccine is available and approved, it would be safe” and a higher proportion (87.8%; p < 0.01) of those who disagreed indicated acceptance of the COVID-19 vaccine for their children than those who neither agreed nor disagreed (53.4%; p < 0.01), and those who agreed (45.8%; p < 0.01) (Table 4). More than half (53.5%) of the respondents disagreed that “COVID-19 vaccine is the most likely way to stop this pandemic” and most (85.1%; p < 0.01) of those who disagreed indicated acceptance of the COVID-19 vaccine for their children than those who neither agreed nor disagreed (65.0%; p < 0.01), and those who agreed (53.8%; p < 0.01). With reference to the complication of the COVID-19 vaccine, less than half (48.2%) of the respondents disagreed with the statement, “The best way to avoid the complications of COVID-19 is by being vaccinated”. The majority (84.5%) of those who disagreed indicated acceptance of the COVID-19 vaccine for their children (p < 0.01) than those who neither agreed nor disagreed (67.4%; p < 0.01) and those who agreed (55.0%; p < 0.01). About two-fifth (40.5%) of the respondents disagreed with the statement “The available vaccines are not effective for curtailing the spread of the virus”.

**Table 4:** Beliefs about COVID-19 vaccines.

		Acceptability of COVID-19 vaccine for children		
Variables	N = 415 n (%)	No n (%)	Yes n (%)	p-value
**Once the vaccine is available and approved, it would be safe**				
Disagree	255 (61.4)	31 (12.2)	224 (87.8)	0.000***
Neither agree nor disagree	88 (21.2)	41 (46.6)	47 (53.4)	
Agree	72 (17.3)	39 (54.2)	33 (45.8)	
**COVID-19 vaccine is the most likely way to stop this pandemic**				
Disagree	222 (53.5)	33 (14.9)	189 (85.1)	0.000***
Neither agree nor disagree	100 (24.1)	35 (35.0)	65 (65.0)	
Agree	93 (22.4)	43 (46.2)	50 (53.8)	
**The best way to avoid the complications of COVID-19 is by being vaccinated**				
Disagree	200 (48.2)	31 (15.5)	169 (84.5)	0.000***
Neither agree nor disagree	135 (32.5)	44 (32.6)	91 (67.4)	
Agree	80 (19.3)	36 (45.0)	44 (55.0)	
**The available vaccines are not effective for curtailing the spread of the virus**				
Disagree	168 (40.5)	39 (23.2)	129 (76.8)	0.289
Neither agree nor disagree	177 (42.7)	49 (27.7)	128 (72.3)	
Agree	70 (16.9)	23 (32.9)	47 (67.1)	
**I believe the vaccine could cause miscarriage in pregnant women**				
Disagree	171 (41.2)	26 (15.2)	145 (84.8)	0.000***
Neither agree nor disagree	151 (36.4)	64 (42.4)	87 (57.6)	
Agree	93 (22.4)	21 (22.6)	72 (77.4)	
**I believe the vaccine could cause a weakened immune system after administration**				
Disagree	213 (51.3)	41 (19.2)	172 (80.8)	0.001**
Neither agree nor disagree	138 (33.3)	52 (37.7)	86 (62.3)	
Agree	64 (15.4)	18 (28.1)	46 (71.9)	
**I believe the administration of the vaccine might be a means to capture an individual’s biodata**				
Disagree	220 (53.0)	43 (19.5)	177 (80.5)	0.001**
Neither agree nor disagree	135 (32.5)	51 (37.8)	84 (62.2)	
Agree	60 (14.5)	17 (28.3)	43 (71.7)	
**I am of the notion that physiological/natural immunity is better compared to vaccine-induced immunity**				
Disagree	153 (36.9)	21 (13.7)	132 (86.3)	0.000***
Neither agree nor disagree	106 (25.5)	28 (26.4)	78 (73.6)	
Agree	156 (37.6)	62 (39.7)	94 (60.3)	
**I believe in God’s protection against COVID-19 compared to the vaccine’s protection**				
Disagree	235 (56.6)	46 (19.6)	189 (80.4)	0.001**
Neither agree nor disagree	84 (20.2)	30 (35.7)	54 (64.3)	
Agree	96 (23.1)	35 (36.5)	61 (63.5)	
**I can relate to the fact that the vaccine is likened unto the mark of the beast**				
Disagree	316 (76.1)	63 (19.9)	253 (80.1)	0.000***
Neither agree nor disagree	73 (17.6)	34 (46.6)	39 (53.4)	
Agree	26 (6.3)	14 (53.8)	12 (46.2)	
**I believe the vaccine programming may be likened to the new world order**				
Disagree	279 (67.2)	50 (17.9)	229 (82.1)	0.000***
Neither agree nor disagree	94 (22.7)	38 (40.4)	56 (59.6)	
Agree	42 (10.1)	23 (54.8)	19 (45.2)	

p < .1*, p < .05**, p < .001***

With regards to vaccines causing miscarriages, about 41% of the respondents disagreed with the statement “I believe the vaccine could cause miscarriage in pregnant women”, and a higher proportion (84.8%; p < 0.01) of those who disagreed indicated acceptance of the COVID-19 vaccine for their children than those who agreed (77.4%; p < 0.01), and those who neither agreed nor disagreed (57.6%; p < 0.01). Slightly more than half (51.3%) of the parents and guardians disagreed with the statement “I believe the vaccine could cause a weakened immune system after administration”. Among the proportion of those who disagreed with the statement, about eight out of ten (80.8%; p < 0.05) indicated acceptance of the COVID-19 vaccine for their children than those who agreed (71.9%; p < 0.05), and those who neither agreed nor disagreed (62.3%; p < 0.05). In addition, the majority (53.0%) of the respondents disagreed with the statement “I believe the administration of the vaccine might be a means to capture an individual’s biodata”. Most (80.5%; p < 0.05) of the respondents who disagreed with the statement indicated acceptance of the COVID-19 vaccine for their children than those who agreed (71.7%; p < 0.05), and those who neither agreed nor disagreed (62.2%; p < 0.05).

About 36.9% of the respondents disagreed with the statement “I am of the notion that physiological/natural immunity is better compared to vaccine-induced immunity” and most (86.3%; p < 0.01) of those who disagreed with the statement indicated acceptance of the COVID-19 vaccine for their children than those who neither agreed nor disagreed (73.6%; p < 0.01), and those who agreed (60.3%; p < 0.01). More than half (56.6%) of the respondents disagreed with the statement “I believe in God’s protection against COVID-19 compared to vaccine’s protection” and eight out of ten (80.4%; p < 0.05) of those who disagreed indicated acceptance of the COVID-19 vaccine for their children than those who neither agreed nor disagreed (64.3%; p < 0.05), and those who agreed (63.5%; p < 0.05). The majority (76.1%) of the respondents disagreed with the statement “I can relate the fact that the vaccine is likened unto the mark of the beast” and most (80.1%; p < 0.01) of those who opposed indicated acceptance of the COVID-19 vaccine for their children than those who neither agreed nor disagreed (53.4%; p < 0.01), and those who agreed (46.2%; p < 0.01).

Furthermore, about 67% of the respondents disagreed with the statement “I believe the vaccine programming may be likened to the new world order,” and a higher proportion (82.1%; p <0.01) of those who disagreed indicated their acceptance of the COVID-19 vaccine for their children than those who neither agreed nor disagreed (59.6%; p < 0.01), and those who agreed (45.2%; p < 0.01).

### Factors associated with parents’ and guardians’ acceptability of COVID-19 vaccine for their children

Only variables with a *p* < 0.05 in bivariate analysis were integrated into the model. Table 5 shows the factors associated with parents’ and guardians’ acceptability of the COVID-19 vaccine for their children. The results of the study showed that factors including education of parents and guardians, belief that COVID-19 be cured, and beliefs about COVID-19 vaccines (“once the vaccine is available and approved, it would be safe”, “the best way to avoid the complications of COVID-19 is by being vaccinated”, “I am of the notion that physiological/natural immunity is better compared to vaccine-induced immunity” and “I believe the vaccine programming may be likened to the new world order”) were significantly associated with parents’ and guardians’ acceptability of COVID-19 vaccine for their children.

**Table 5:** Logistic regression model of acceptability of COVID-19 vaccine for children.

	Acceptability of COVID-19 vaccine for children R^2^ = 0.412		
Variables	Adjusted Odds Ratio (AOR)	95% CI	p-value
**Education of parent/guardian**			
Less than SHS (RC)			
SHS	0.390	0.153–0.989	0.047**
Undergraduate	0.545	0.219–1.354	0.191
**Can COVID-19 be cured**			
Yes (RC)			
No	0.536	0.307–0.935	0.028**
**Once the vaccine is available and approved, it would be safe**			
Disagree (RC)			
Neither agree nor disagree	0.283	0.132–0.607	0.001***
Agree	0.248	0.111–0.556	0.001***
**COVID-19 vaccine is the most likely way to stop this pandemic**			
Disagree (RC)			
Neither agree nor disagree	0.994	0.448–2.207	0.989
Agree	0.533	0.245–1.161	0.113
**The best way to avoid the complications of COVID-19 is by being vaccinated**			
Disagree (RC)			
Neither agree nor disagree	0.369	0.175–0.778	0.009**
Agree	0.487	0.218–1.085	0.078*
**I believe the vaccine could cause miscarriage in pregnant women**			
Disagree (RC)			
Neither agree nor disagree	0.529	0.260–1.073	0.078*
Agree	1.288	0.539–3.078	0.568
**I believe the vaccine could cause weakened immune system after administration**			
Disagree (RC)			
Neither agree nor disagree	1.262	0.628–2.537	0.513
Agree	2.137	0.853–5.351	0.105
**I believe the administration of the vaccine might be a means to capture an individual’s biodata**			
Disagree (RC)			
Neither agree nor disagree	0.788	0.406–1.529	0.481
Agree	1.253	0.504–3.114	0.628
**I am of the notion that physiological/natural immunity is better compared to vaccine-induced immunity**			
Disagree (RC)			
Neither agree nor disagree	0.524	0.241–1.139	0.103
Agree	0.278	0.138–0.559	0.000***
**I believe in God’s protection against COVID-19 compared to the vaccine’s protection**			
Disagree (RC)			
Neither agree nor disagree	0.608	0.296–1.246	0.174
Agree	0.858	0.421–1.749	0.674
**I can relate to the fact that the vaccine is likened unto the mark of the beast**			
Disagree (RC)			
Neither agree nor disagree	0.800	0.322–1.992	0.632
Agree	0.833	0.234–2.965	0.778
**I believe the vaccine programming may be likened unto the new world order**			
Disagree (RC)			
Neither agree nor disagree	1.013	0.419–2.450	0.976
Agree	0.334	0.118–0.944	0.039**

RC: Reference Category, *p* < .1*, *p* < .05**, *p* < .001***

Parents and guardians with SHS education were less likely (AOR = 0.390; CI 95% 0.153–0.989; p = 0.047) to accept the COVID-19 vaccine for their children than those with less than Senior High School. Parents and guardians who did not agree that COVID-19 could be cured had a lower likelihood (AOR = 0.536; CI 95% 0.307–0.935; p = 0.028) of accepting the COVID-19 vaccine for their children than those who agreed that COVID-19 could be cured. Parents and guardians who neither agreed nor disagreed (AOR = 0.283; CI 95% 0.132–0.607; p = 0.001) and those who agreed (AOR = 0.248; CI 95% 0.111–0.556; p = 0.001) to the statement “once the vaccine is available and approved, it would be safe” were less likely to accept COVID-19 vaccine for their children than those who disagreed to the statement. In addition, parents and guardians who neither agree nor disagree with the statement “the best way to avoid the complications of COVID-19 is by being vaccinated” were less likely (AOR = 0.369; CI 95% 0.175–0.778; p = 0.009) to accept COVID-19 vaccine for their children compared to those who disagreed to the statement. Also, parents and guardians who agreed to the statement “I am of the notion that physiological/natural immunity is better compared to vaccine-induced immunity” had a lower likelihood (AOR = 0.278; CI 95% 0.138–0.559; p = 0.000) of accepting the COVID-19 vaccine for their children than those who disagreed to the statement. Finally, parents and guardians who agreed to the statement “I believe the vaccine programming may be likened to the new world order” were less likely (AOR = 0.334; CI 95% 0.118–0.944; p = 0.039) to accept the COVID-19 vaccine for their children than those who disagreed to the statement.

## Discussion

This study examined parents’ and guardians’ acceptability of the COVID-19 vaccine for their children under age 18 in light of the limited data on the issue in sub-Saharan Africa since most studies have focused on adults’ acceptability of the vaccine. The current study indicates that most parents and guardians (73.3%) intended to vaccinate their children against COVID-19 if the Government of Ghana provided an approved vaccine. The high acceptability of the COVID-19 vaccine for children found in this study is comparable to other online surveys carried out by Zhang et al.’s [23] study in China and that of Kelly et al. [24] in the United States, which reported that about 73% of parents, would allow their children to be vaccinated against COVID-19, respectively. However, in Zambia, Carcelen et al. [27] reported that 92% of caregivers intended to vaccinate their children against COVID-19. Du et al.’s [19] study in China also found that 91.6% of women in their reproductive ages intended to vaccinate their children against COVID-19.

The prevalence of acceptability of COVID-19 vaccination for children found in this study is higher than the prevalence found in other countries. For instance, a study in Italy among parents and guardians of children less than 18 years found that 60.4% of parents and guardians intended to vaccinate their children against COVID-19 [21]. Another study in England among parents and guardians found that 48.2% of parents and guardians had the intention to vaccinate their children against COVID-19 [25].

Studies have found that the main reason for parents’ intention to vaccinate their children is to protect them [30–33]. Therefore, parents are more likely to vaccinate their children against COVID-19 if they already receive other national immunization vaccines [27, 34, 35]. On the other hand, doubts about a vaccine’s safety and effectiveness are mainly cited as concerns for vaccine hesitancy [3, 20, 25, 36]. Similarly, in this study, parents and guardians who were unwilling to accept the vaccine for their children were mainly concerned about the lack of information on the safety and adverse effect of the vaccine. However, vaccination of children generally has positive health and social impacts, such as reducing deaths, particularly among children under five years of age, better psychological development and learning abilities [18, 37]. This calls for an urgent need for public health practitioners to intensify public communication about the vaccine’s side effects and safety to alleviate parents’ and guardians’ fears.

Educational attainment was the only socio-demographic variable significantly associated with parents’ and guardians’ intention to vaccinate their children. This finding highlights the importance of formal education on health and related behaviours. Some studies have demonstrated a positive association between vaccine acceptance and parental education [38, 39]. However, comparable to other studies in Saudi Arabia [40], China [41], and Turkey [35], we found an inverse relationship between educational level and parents’ intention to vaccinate their children against COVID-19. Hu et al. [41] explained that a higher educational level might increase concern about the safety and quality of the vaccines, hence, the lower likelihood of vaccine acceptance among parents/guardians with secondary or higher educational levels. This would negatively have an impact on COVID-19 vaccination for children in Ghana. With more than half (51.8%) of the Ghanaian population with secondary or higher education [42], there is a need for public health practitioners to continuously sensitise the general public, particularly, the educated about the safety and quality of the COVID-19 vaccines to reduce vaccine hesitancy for children.

Research has established that the perceived cure for a health condition influences individuals’ care-seeking behaviour [43, 44]. Individuals who perceive that their health conditions can be cured actively engage in cure seeking and vice versa. This study found that parents and guardians who perceived that COVID-19 could not be cured were less likely to report the intention to vaccinate their children against COVID-19 compared to those who perceived that it could be cured. It is understandable why these parents and guardians believed that vaccinating their children was unnecessary because they perceived that COVID-19 could not be cured. Therefore, public health practitioners are urged to increase awareness among parents, guardians, and the general public of the advantages of vaccinating against COVID-19.

Generally, beliefs surrounding vaccines may significantly affect their hesitancy or acceptance. This was evident in our study. We found that four variables that measured beliefs about COVID-19 vaccines significantly predicted parents’ and guardians’ intention to accept the vaccine for their children. Parents who perceived that an approved vaccine would be safe were less likely to report the intention to vaccinate their children against COVID-19. The reason could be that beliefs do not necessarily translate to action. Probably, the parents and guardians perceive the COVID-19 vaccine to be safe but may not see the need and relevance of allowing their children to vaccinate due to the side effects and other related issues. This finding is contrary to Carcelen et al.’s [27] study in Zambia, which found that caregivers who perceived that the COVID-19 vaccine would be safe were more likely to report the intention to vaccinate their children than those who believed otherwise.

This study also found that parents and guardians who were neutral about the fact that the best way to avoid complications of COVID-19 is by being vaccinated were less likely to vaccinate their children. Since these parents and guardians are indifferent about vaccination being the best way to prevent COVID-19 complications, they do not see the need to vaccinate their children against it. Convincing these parents and guardians that the best way to avoid complications of COVID-19 is through vaccination would help reduce vaccine hesitancy.

Furthermore, parents and guardians who affirmed that physiological/natural immunity is better than vaccine-induced immunity were less likely to accept vaccination for their children. A possible explanation is that both natural and vaccine-induced immunities are effective against the virus [45]. Therefore, the perception that physiological/natural immunity is better may discourage an individual from accepting vaccine-induced immunity.

Parents and guardians who believe the vaccine programming may be likened to the new world order are less likely to accept covid-19 vaccination for their children. Misinformation about COVID-19 vaccines has been one of the main contributors to the unwillingness of individuals to accept the vaccine [46–48].

### Limitations

This study has some limitations that are worth noting. First, the study population is non-representative of Ghana’s population since we used convenient and snowballing sampling techniques. Second, all respondents who partook in the study had formal education and internet access. Based on these limitations, the findings of this study should be interpreted with caution. Third, the child’s age and whether or not the parent and guardian have been vaccinated against COVID-19 can influence the intention to vaccinate their children against COVID-19. However, these characteristics were not included in the study.

Moreover, this study was cross-sectional, so causality cannot be established. Finally, the link for the online survey was shared on WhatsApp, Meta, and Twitter platforms, and it was anonymous, so a respondent could complete the survey more than once. Despite these limitations, this study is among the few studies conducted on parents’ and guardians’ intention to vaccinate their children against COVID-19 in sub-Saharan Africa, including Ghana. Therefore, findings from this study would contribute to the literature on vaccine hesitancy in sub-Saharan Africa.

## Conclusions

Findings from this study indicated that most Ghanaian parents and guardians are willing to accept COVID-19 vaccines for their children. However, they have concerns about the inadequate data about the safety of COVID-19 vaccines and their adverse effects. In addition, the acceptability of COVID-19 vaccines for their children was influenced by their educational level, perception about the cure of COVID-19, and beliefs about COVID-19 vaccines. Therefore, to increase the acceptability of COVID-19 vaccines for children, public health practitioners should consider these factors in designing and implementing COVID-19 vaccination campaigns. Specifically, public health practitioners should periodically sensitise parents, guardians, and the general public on the benefits and side effects of COVID-19 vaccines. Also, they should provide regular and up-to-date information about the safety of COVID-19 vaccines to allay the fears of parents and guardians. In addition, further studies should use a mixed study approach to understand vaccine hesitancy comprehensively.

## Supporting information

S1 Questionnaire Questionnaire for parents’ and guardians’ acceptability of COVID-19 vaccination for children in Ghana. (DOCX)

S1 Appendix Dataset. (XLSX)
