# Supplementary material for: Parents’ and guardians’ acceptability of COVID-19 vaccination for children in Ghana: An online survey
Source: PLoS One. 2022 Aug 29;17(8):e0272801. doi: 10.1371/journal.pone.0272801 (PMC9423663; doi:10.1371/journal.pone.0272801)
Supplement: S1 Questionnaire — (DOCX) [file pone.0272801.s001.docx]

**Questionnaire for** **Parents’ and guardians’ acceptability of COVID-19 vaccination for children in Ghana**

**Socio-demographic characteristics**

1. What is your age?
2. < 20
3. 20 – 29
4. 30 – 39
5. 40 – 49
6. 50 – 59
7. 60 and above
8. What is your sex?
9. Male
10. Female
11. What is your religious affiliation?
12. Christian
13. Islam
14. Traditionalist
15. No Religion
16. Other (specify):­­­­­­­­­­­­­­­­­­­­­­­­­­________________
17. What is your highest level educational attained?
18. No formal education
19. Pre-school
20. Primary
21. JSS/JHS
22. Middle
23. SSS/SHS
24. Undergraduate
25. Postgraduate
26. What is your marital status?
27. Never married
28. Informal/living together
29. Married
30. Separated
31. Divorced
32. Widowed
33. What is your employment status?
34. Currently employed
35. Not currently employed
36. What is your current place of residence?

Suburb/locality: __________________________

District: _________________________________

Region: __________________________________

1. How many years have you stayed in your current suburb/locality? __________________
2. In general, would you say that you/your household has:
3. More money than you need
4. Just enough money
5. Less money than you need
6. How many children (below 18 years) do you have? _____________________
7. How many of your children are males: _________________
8. How many of your children are females:________________

**COVID-19 experience**

1. Have you ever had a COVID-19 test? ***If No skip to Q. 15***
2. Yes
3. No
4. If yes Q. 13, what was the result of the test? ***Skip to Q. 16***
5. Positive
6. Negative
7. Don’t Know / I did not receive the result
8. If no to Q. 13, would you like to have a COVID-19 test?
9. Yes
10. No
11. Don’t Know/Can’t tell
12. If yes to Q. 15, do you know where to have a COVID-19 test?
13. Yes
14. No
15. If no to Q. 15, why would you not want to be tested?

________________________________________________________________________________________________________________________________________________

1. Has a member of your household been diagnosed with COVID-19?
2. Yes
3. No
4. If yes to Q. 18, what is your relationship with this person?
5. Spouse
6. Child (Son/Daughter)
7. House help
8. Other (specify)________________________________
9. Has any of your relatives been diagnosed with COVID-19?
10. Yes
11. No
12. Don’t Know/Can’t tell
13. Has any one of your friends been diagnosed with COVID-19?
14. Yes
15. No
16. Don’t know/Can’t tell
17. Has any one of your neighbours been diagnosed with COVID-19?
18. Yes
19. No
20. Don’t know/Can’t tell
21. What is your child’s chance of getting COVID-19 at home?
22. Small
23. Moderate
24. Great
25. No risk at all
26. Please explain your response in Q. 23

____________________________________________________________________________________________________________________________________________________________

1. What is your child’s chance of getting COVID-19 in your community/neighbourhood?
2. Small
3. Moderate
4. Great
5. No risk at all
6. Please explain your response in Q. 25

____________________________________________________________________________________________________________________________________________________________

1. What is your child’s chance of getting COVID-19 at school?
2. Small
3. Moderate
4. Great
5. No risk at all
6. Please explain your response in Q. 27

____________________________________________________________________________________________________________________________________________________________

1. Have you heard of COVID-19 vaccine? ***If No Skip to Q 33***
2. Yes
3. No
4. If yes to Q. 29, where did you hear of it? ***Select all sources that applies***

| TV |  |
| --- | --- |
| Radio |  |
| Internet |  |
| Friends/Relatives |  |
| Newspaper/Magazine |  |
| Workplace |  |
| Health workers |  |
| Church/Mosque |  |
| Pamphlets/Posters |  |
| Other (specify) |  |

1. Has news of government’s decision to provide COVID-19 vaccine changed your adherence to COVID-19 prevention protocols?
   1. Yes
   2. No
2. If yes in Q. 31, what did you do? ________________________________________________________________________________________________________________________________________________
3. Is it possible for a healthy looking person to have COVID-19?
   1. Yes
   2. No
   3. Don’t know/Can’t tell
4. Can COVID-19 be cured?
   1. Yes
   2. No
   3. Don’t know/Can’t tell

**Beliefs about COVID-19 vaccines**

**Kindly indicate the extent to which you agree or disagree with each statement on beliefs about COVID-19 vaccines**

|  |  | **Strongly disagree** | **Disagree** | **Neither agree nor disagree** | **Agree** | **Strongly agree** |
| --- | --- | --- | --- | --- | --- | --- |
|  | Once the vaccine is available and approved, it would be safe |  |  |  |  |  |
|  | COVID-19 vaccine is the most likely way to stop this pandemic |  |  |  |  |  |
|  | The best way to avoid the complications of COVID-19 is by being vaccinated |  |  |  |  |  |
|  | The available vaccines are not effective for curtailing the spread of the virus |  |  |  |  |  |
|  | I believe the vaccine could cause miscarriage in pregnant women |  |  |  |  |  |
|  | I believe the vaccine could cause weakened immune system after administration |  |  |  |  |  |
|  | I believe the administration of the vaccine might be a means to capture individual’s biodata |  |  |  |  |  |
|  | I am of the notion that physiological/natural immunity is better compared to vaccine induced immunity |  |  |  |  |  |
|  | I believe in God’s protection against COVID-19 compared to vaccine’s protection |  |  |  |  |  |
|  | I can relate the fact that the vaccine is likened unto the mark of the beast |  |  |  |  |  |
|  | I believe the vaccine programming may be likened unto the new world order |  |  |  |  |  |

**Intention to vaccinate child/children**

1. Have you heard about the intended COVID-19 vaccination for all Ghanaians?
2. Yes
3. No
4. Don’t know
5. If an approved COVID-19 vaccine became available, would you allow your child/children to be injected/vaccinated?
6. Yes
7. No
8. Don’t know
9. If No, what is the reasons for your unwillingness to allow your child/children to receive COVID-19 vaccine?
10. Inadequate data about the safety of the new vaccine
11. A concern on adverse effects of the vaccine
12. A concern on vaccine being ineffective
13. Prior adverse reaction to any vaccine
14. I am against vaccines in general
15. A concern of acquiring COVID-19 infection from the vaccine itself
16. I perceive my child not to be at considerable risk of developing complications if he/she is infected with COVID-19
17. I perceive my child not at elevated risk to acquire COVID-19 infection
18. My child already had COVID-19 infection
19. Vaccine administration is painful or inconvenient
20. Vaccination is a violation of my religious doctrines
21. Other (specify)___________________________
22. Among the reasons for Q31, what is the main reason for your unwillingness to allow your child/children to receive COVID-19 vaccine?
23. Inadequate data about the safety of the new vaccine
24. A concern on adverse effects of the vaccine
25. A concern on vaccine being ineffective
26. Prior adverse reaction to any vaccine
27. I am against vaccines in general
28. A concern of acquiring COVID-19 infection from the vaccine itself
29. I perceive my child not to be at considerable risk of developing complications if he/she is infected with COVID-19
30. I perceive my child not at elevated risk to acquire COVID-19 infection
31. My child already had COVID-19 infection
32. Vaccine administration is painful or inconvenient
33. Vaccination is a violation of my religious doctrines
34. Other (specify)___________________________
35. Are there any personal concerns in relation to COVID-19?

_____________________________________________________________________________

**Thank you**
